# ‘Obviously, because it’s a tear it won’t necessarily mend itself’: a qualitative study of patient experiences and expectations of treatment for a meniscal tear

**DOI:** 10.1136/bmjopen-2024-088656

**Published:** 2025-01-14

**Authors:** Imran Ahmed, Chetan Khatri, Fatema G Dhaif, Charles E Hutchinson, Nicholas Parsons, Andrew James Price, Sophie Staniszewska, Andrew Metcalfe

**Affiliations:** 1 University Hospital Coventry and Warwickshire, Coventry, UK; 2 Clinical Trials Unit, University of Warwick, Coventry, UK; 3 Warwick Medical School, University of Warwick, Coventry, UK; 4 Nuffield Department of Orthopaedics, Rheumatology and Musculoskeletal Sciences, University of Oxford, Oxford, UK; 5 Warwick Clinical Trials Unit, University of Warwick, Warwick Medical School, Coventry, UK; 6 Trauma and Orthopaedics, University Hospitals Coventry and Warwickshire NHS Trust, Coventry, UK

**Keywords:** Knee, QUALITATIVE RESEARCH, SURGERY

## Abstract

**Abstract:**

**Objectives:**

There is a paucity of qualitative research exploring the patient experience of living with a meniscal tear, vital to effective patient management. The aim of this study was to explore the experiences and expectations of treatment of patients aged 18–55 years with a meniscal tear of the knee.

**Design:**

Qualitative study involving semistructured interviews.

**Setting:**

Participants were selected from nine secondary care centres in England.

**Participants:**

10 participants diagnosed with a meniscal tear were recruited from the Meniscal Tear Outcome cohort study using a purposive sampling strategy until data saturation was achieved. Semistructured interviews were conducted between April and May 2021 and thematic analysis was used to identify key patient experiences.

**Results:**

Themes identified relate to the broad areas of symptoms, clinical consultation and experience of treatment. Meniscal tears have a profound impact on pain and many patients experience effects on their family and financial life in addition to physical symptoms. Participants expected most of their management to occur in secondary care and most thought surgery would be a definitive treatment, while they believed the effectiveness of physiotherapy could not be guaranteed as it would not fix the physical tear.

**Conclusion:**

This study is one of the first to explore patient experiences of a meniscal tear and their perceptions of treatment options. Patient experiences and perceptions are important for clinicians to understand in order to provide the best possible care. It is important to elicit these experiences, listen to the patient, discuss their perspectives and build these experiences and expectations into clinical care.

**Trial registration number:**

ISRCTN11534691.

**STRENGTHS AND LIMITATIONS OF THIS STUDY**

- Participants were identified using a purposive sampling method based on age, sex and type of treatment, that is, operative or non-operative.
- Peer coding was performed.
- Interviews were conducted virtually due to the COVID-19 pandemic.
- Participants recruited from secondary care may not reflect patient experiences who are managed solely in primary care.

## Introduction

 Meniscal tears are a very common knee pathology which can be separated in traumatic and degenerative meniscal tears.^1^ This differentiation could be based on injury mechanism or the presence of degenerative changes within the knee.^1^,^3^ There is clear high-quality evidence suggesting no increased benefit of surgery in those patients with a meniscal tear and osteoarthritis in the knee or in older patients. However, there is uncertainty regarding the effectiveness of surgery in younger patients or those without arthritis.

National and international bodies including the British Association for Surgery of the Knee and the European Society of Sports Traumatology, Knee Surgery and Arthroscopy have produced treatment guidelines based on expert consensus meetings.^4 5^ However, the production of these guidelines did not involve patients or public members, unlike other guidelines on degenerative knee conditions as a whole.^6^ In addition, recent randomised controlled trials have demonstrated that operative treatment may not be superior to non-operative treatments.^7^ However, over 25% of patients crossed over into the non-operative arm. The authors of one study also highlighted that the true difference in change may be in favour of the surgery group.^7^ Further research is needed to explore patient views on current treatment pathways in order to understand why patients cross over to operative interventions.

There has been an increase in the availability of qualitative research for musculoskeletal pathologies such as hip and ankle fracture.^8 9^ There are few qualitative studies exploring the patient experience for knee pathologies, such as meniscal tears.^10 11^ A previous systematic review found two qualitative studies reporting the experiences of patients with a meniscal tear. The key themes identified in these studies were that patients perceived surgery was a quicker and more definitive treatment and the patient believed that findings on imaging was a key driver in the decision making process.^12^ There is a need to for further qualitative studies to identify patient experiences of meniscal tears and their expectations of treatment in order to inform clinicians on patient views on investigations and treatment preferences.

### Aim

The aim of this study is to explore the experiences and expectations of treatment of patients aged 18–55 years with a meniscal tear of the knee.

## Materials and methods

A semistructured interview study was undertaken with participants from the Meniscal Tear Outcome (METRO) study cohort.^13^

### Participant selection

Participants were selected from the METRO cohort study, a national multicentre prospective cohort study exploring the outcomes of patients aged 18–55 years with a meniscal tear presenting to secondary care.^13^

The following eligibility criteria were applied.

#### Inclusion criteria

- Age 18–55 years.
- MRI confirmed meniscal tear.
- Written consent.

#### Exclusion criteria

- Anterior cruciate ligament injury or another ligament injury.
- Associated intra-articular fracture.
- Previous knee surgery.
- Previous entry into the METRO cohort study.
- Participant unable to undertake study procedures.
- Established radiographic knee osteoarthritis.

Participants were identified using a purposive sampling method based on age, sex and type of treatment, that is, operative or non-operative. The lead author (IA) approached participants and provided them with a participant information sheet and consent form. The aim of purposive sampling was to achieve a satisfactory mix of patient ages, sex and treatment options. At the time of interview, all participants had already received treatment by a physiotherapist and had been reviewed in secondary care by an orthopaedic surgeon. Once a participant agreed to take part, the interview time was arranged and the interview was conducted. Once data saturation was achieved, participants who had agreed to take part but had not completed their interview were contacted informing them that they were no longer needed.

A total of 10 participants were interviewed in April and May 2021 (demographic details in table 1).

**Table 1:** Baseline characteristics of study participants

Participant number	Sex	Age (range)	Mechanism of injury	Months since recruitment	Management of meniscal tear	Duration of knee pain (months)	Duration of interview (minutes)
1	Male	40–45	Does not recall a specific event	22	Non-operative	12	38
6	Female	50–55	Fall during walk	22	Operative	14	28
26	Female	50–55	Does not recall a specific event	19	Non-operative	24	39
39	Female	50–55	Foot caught in pothole	18	Non-operative	3	59
55	Male	45–50	Running	16	Non-operative	2	38
80	Male	40–45	Does not recall a specific event	14	Non-operative	12	28
100	Male	30–35	Does not recall a specific event	13	Operative	24	38
130	Male	20–25	Gym training	6	Operative	4	47
144	Male	45–50	Does not recall a specific event	3	Non-operative	30	23
906	Male	35–40	Does not recall a specific event	6	Operative	5	40

All interviews were performed virtually based on advice from the patient and public involvement group. Participant numbers relate to their study number from the METRO cohort study. Age was presented as a range to preserve the anonymity of the participants.

### Data collection

Data were collected using semistructured interviews method with a predetermined interview schedule to ensure key questions were asked but there was an opportunity for participants to raise issues of importance. The interview schedule was designed following a discussion with senior members of the trial team (IA, AM and SS) and the METRO patient and public involvement group. The interview schedule consisted of four parts: experiences of meniscal tear, seeking clinical help, current outcome scores and future research ideas. The interview schedule can be seen in full in online supplemental appendix 1.

Due to the COVID-19 pandemic, participants were given the option to undertake interviews via telephone or videoconferencing. Interviews were recorded using an encrypted Dictaphone prior to transcription. Transcription was provided by a secure third party (Appen Transcription services, New South Wales, Australia).

### Reflexivity

IA approached all participants to consent them for the interview study and conducted all the interviews. IA is an orthopaedic registrar and PhD student but introduced himself as a researcher from Warwick Clinical Trials Unit to prevent participants making assumptions which might influence the direction of the interview and ensure they feel comfortable discussing their experiences of the treatment pathway without feeling pressure of judgement from a clinician. Additionally, it was made clear that IA was not involved in the treatment process and could not influence treatment decisions.

It is important to note that IA may have subconscious biases towards surgery given his background, therefore, the following mitigations prevented the role of the interviewer from influencing the direction and content of the interviews.

Using semistructured interviews helped reduce the risk of steering the interview completely towards surgery. A prespecified interview guide ensured that the interview addressed the aims of the study and allowed the interviews to be performed from a neutral position in terms of treatment preference. After each interview, reflective notes were produced in order to highlight any issues which arose during the interviews and to evaluate any assumptions and whether they influenced the interview.

### Context

Participants were approached for interviews in March 2021. All interviews took place in April and May 2021. The METRO cohort study recruitment period ran from April 2019 to September 2021. Due to the cross-sectional nature of this interview study, some patients had been recruited from the very start of their treatment in secondary care whereas others may have completed their treatment journey.

### Data analysis

Data analysis was undertaken using the six-step thematic analysis framework devised by Braun *et al*.^14^ All transcripts were read twice before generating codes. Once all transcripts were read and coded, they were re-read to allow for further clarification of the codes. Subsequently, themes which summarised the codes were generated and reviewed. All interviews were coded by IA, and FGD independently coded four interviews as a means of peer coding. Oversight was provided by senior author SS (A professor who specialises in qualitative research).

Previous studies have found that 94% of high frequency codes were identified in the first 6 interviews and 97% of high frequency codes identified in the first 12 interviews.^15^

After nine interviews, saturation of data was reached with no new themes identified from the 9th and 10th interviews. During the recruitment period, through purposive sampling, 17 participants from the METRO cohort population of 200 were initially invited for interview. As 10 participants agreed to be interviewed, no further participants were invited for interview at that point. These 17 patients were selected based on sex, age, number of months since recruitment, duration of knee pain (months) and management of meniscal tear.

### Patient and public involvement

A study advisory group was created with two patient members. Both members were involved in all stages of this research. The study advisory group provided assistance in the production of the interview schedule and data collection methods. The group felt that following the pandemic patients would prefer telephone interviews rather than face-to-face interviews. The patient members were also involved in data analysis as they reviewed the themes generated and provided an insight into the study authors’ interpretation of the data. Results of this study will also be disseminated by lay summaries available on the METRO social media page.

## Results

10 participants were interviewed prior to data saturation. 13 themes were identified during the data analysis, which were separated into the following three divisions for ease of interpretation: (1) participant experiences of their condition, (2) clinical consultations and (3) treatment.

### Participant experiences of meniscal tear

Meniscal tears appeared to have an immediate impact due to the symptoms experiences, a medium-term impact on family life and occupation and a long-term impact on emotional well-being and financial well-being. Themes identified included:

#### The experience of severe pain

Many participants described the severe pain which led to them seeking medical attention. They also described the impact this pain had on their daily lives in the short term. Participant 55 when asked to describe the pain said *‘I was in agony to be honest, I couldn’t, I couldn’t put any weight on it at all, yes I was in absolute bits and it was swollen, it was definitely swollen I can just remember that and it was really, really uncomfortable.’* Participant 26 described this further by saying *‘The pain, pain, I could hardly walk on both my knees and it was just gradually getting worse and worse. I couldn’t sleep at night for the pain in the back of my, back of my leg. And yeah pain, constant pain 24 hours a day.’*

#### An inability to perform previous activities

Participants described that they were unable to perform activities they previously participated in or had to adjust their activities due to the symptoms of their meniscal tear. Participant 906 mentioned *‘I couldn’t get back upstairs. I couldn’t bear weight on it much at all, so I slept downstairs.’* Participants mentioned having to give up sport and exercise due to the meniscal tear. Participant 80 mentioned ‘*Well sports basically, I can’t do them anymore because of the pressure on my knee, it’s very tender.’* Participant 144 described changing their regular activities because of the injury and described taking up *‘non-impact sports like cycling’*.

#### The wider effects of meniscal tears on work and family life

The impact of meniscal tears affected participants’ wider social circles including relationships with family members and dependents. Participant 100 said *‘I’ve got two young children so it was bathing them and that was a struggle.’* This was similar to participant 80 who mentioned *‘I seem to have a bit of shorter temper with my three year-old son, cause he often jumps but he forgets I’ve got a bad knee and he jumps.’* Participants also described how they had to change careers or take time off work due to their condition. *‘So I'm now sort of a contracts manager. ‘Cause again I wouldn’t wanna be kneeling, kneeling on sites, all day every day on my knees, even now, kneeling isn’t the most comfortable thing.’* Participant 100.

#### The long-term effects on financial and emotional well-being

Participants described the long-term financial impact of meniscal tears and the effect this had on their emotional well-being. Participant 80 said *‘I was getting a little bit worried about (inaudible) you know, like I’ve got bills and a mortgage to pay.’*

### Experiences and expectations of the clinical consultation

#### Participant expectations of imaging and diagnosis

Participants believed that a plain X-ray would identify any issues with the bone and believed that the MRI would provide the more detailed assessment required to diagnose any damage within the knee. Participant 906 when asked about what investigation was expected said *‘I know it was a bit more in depth than an X-ray which is just bone. It’s magnetic resonance imaging that would do, I think, muscles and I, I knew it was more intimate scan.’* Participant 130 was very grateful that they had an MRI to identify a cause for their symptoms mentioning their doctor said *‘Oh well put you in for an MRI just in case’ and then ‘thank God he did.’*

#### The experiences of the initial diagnosis through the participants’ eyes

Participants described identifying a cause of their symptoms either through their own research or following a discussion with clinicians. Participants described often using internet resources to understand or identify a diagnosis. However, one participant described the negative effect this can have by identifying more serious diagnoses. *‘But I don’t want to diagnose myself with Google and before you know it, you’ve got knee cancer.’* (Participant 80). Some felt the injury was due to a specific incident and an increase in activity whereas others did not remember an exact cause of the injury. *‘The tear happened in my basic training as a firefighter. I was bent down with the hose reel in my arms which were about sort of 20, 30 kilos. I sort of stood up and twisted and then pushed sort of on my right leg and that’s when I sort of felt it go.’* (Participant 100).

#### The primary care doctor is the gateway to further treatment

When participants described their expectations and experiences of the primary care doctor, many viewed the primary care service as a gateway into secondary care. Participants expected an examination of their knee during the consultation and were left frustrated if no examination was performed. As mentioned by participant 906 *‘I did expect just a little examination. He actually didn’t even look away from his screen so that, that was a bit annoying ‘cause I knew how much pain I was in with it.’* Once an examination was done most participants expected their treatment to take place in secondary care rather than primary care. They believed the interpretation of imaging and management of meniscal tears would be overseen by specialist orthopaedic doctors. They did not expect their GP to interpret the MRI scan. ‘*GP has got obviously great knowledge but they, I don’t know if they can read MRIs but for stuff like meniscal tear and stuff you know what I mean? Obviously, it’s quite specialised isn’t it, so. But then I think they told me they were going to refer me.’* (Participant 100)

Participants expected further consultations to take place in secondary care. The following theme described their experiences of this further.

#### The experience of the decision-making process: shared or paternalistic approach

Participants described their role in the decision-making process in one of two ways. Some participants trusted the medical team to make the best decision for the participant and did not expect or want a significant role. These participants relied on the clinician taking the role of ‘expert’ in the consultation and accepted their treatment plan. *‘To be fair, I'm not a doctor, and I don't know nothing about body. And to be fair I trust 100% the doctor that I'm going to the hospital.’* (Participant 1)

Other participants attended the consultation with an expectation of a shared decision-making process, whereby the participant and clinician determine the best course of action together and the participant is a co-manager of their condition. This was the opinion of participant 6 who said *‘It was completely down to me. He turned round and said, you know, he goes, ‘Anything you want to know I’ll tell you, but,’ he goes, ‘at the end of the day it’s got to be your choice to whether you say you want me to operate, you carry on as you are, or you try physio.’*

#### Patient experiences and perceptions of continuity of care

Participants who saw the same clinician, built rapport and felt they were being treated as an individual. Participant 55 was aware of limited consultation time and wanted to maximise the impact of consultations on treatment success, therefore, they felt frustrated repeating themselves. *‘Going from appointment to appointment, I’d feel rather than the like doctors, I dunno, checking my notes that I assume there were, I’d have to kind of go through the rigmarole of like explaining everything again. Especially when you’re, you’ve got these specialist appointments and they’re only like 20 minutes long. That was actually a point of frustration.’* Regarding physiotherapy consultations, one participant felt weekly physiotherapy was insufficient as they could progress well at home and often wanted to progress to the next stage of their treatment sooner or felt they needed reassurance they were performing exercises correctly. *‘Probably all week you can do the exercises wrong and only one day you can do the proper exercises.’* (Participant 1)

### Participant experience and expectation of treatment

The following themes were all related to participant views on operative and non-operative treatment for meniscal tears.

#### Surgery is often seen as the definitive treatment

Many participants believed that surgery was the definitive solution for a meniscal tear and it would provide the fastest and most certain way of returning to their premorbid function. When discussed further, participants believed that the meniscus would not repair itself and would require surgery to correct it.

Obviously because it’s a tear it wont necessarily mend itself. (Participant 55)

The meniscus can be broken and it will be a, let’s say a, a, a routine operation to, to adjust the meniscus. And after that your life can be, let’s say, back to normal. (Participant 1)

Well, I think if you’d sewn up the actual tear and actually then its all put together properly.’ Leading to you getting back to normal ‘presumably quicker. (Participant 39)

Participants also felt that surgery was the logical next step from physiotherapy and that in some cases physiotherapy was delaying surgery.

Surely if I have to keep going back for physio or if it happens again, it happens again, you know what if, you know, if that’s, if that’s gonna have a longer impact then surely, yes an operation is not cheap but I think that’s kind of a known fix and it works. (Participant 55)

Participant 55 also mentioned how their views on surgery was formed through discussion with peers and observing the results they had. They *said ‘yeah I had spoken to somebody work had a meniscal tear, he’s a little bit, he’s younger than me and he had, he had his fixed.’ ‘He had his operation and within probably what, I dunno three months or whatever he was back playing golf, running doing everything he, he was kind of, everything he was doing previously.’* This again highlighted the strong views participants had regarding the impact of surgery.

#### ‘Letting nature take its course’: the arguments against surgery

Some participants believed surgery was invasive and should only be the last resort when other treatments have failed. Others believed that the symptoms would naturally improve and did not want to resort to surgery as a treatment. Additionally, some participants did not want an operation due to the financial implications it may have as they may be off work recovering from the operation.

I was getting a little bit worried about (inaudible) you know, like I’ve got bills and a mortgage to pay. (Participant 80)

I said financially it would have been a, it would have been a drain. As I said work I had a certain amount of leave I could take. (Participant 100)

#### The participant perspective on meniscal repair versus removal

Participants discussed their opinions on meniscal repair versus removal: the two mainstays of surgical management. Participants accepted that meniscal repair was likely to protect and preserve the knee cartilage, however, some felt the rehabilitation would have a negative impact on looking after children or returning to work.

So obviously I, I would, I told them at the time, I prefer a removal which, I think they were a bit like, “Oh obviously there’s a lot of a lot else goes with that and obviously life, lifespan of your knee, obviously if it is removed, isn’t great.” But like I say, obviously time, time off work with a young family and stuff I couldn’t have, I couldn’t have had 12 weeks in the full cast, obviously with the repair.’ ‘For my knee health, I know obviously repair would have been a better option. But yeah obviously the removal, the removal for my circumstance and for me was the route I preferred. (Participant 100)

#### Success was not guaranteed with physiotherapy

Although participants felt that surgery provided a definitive fix, many participants believed physiotherapy did not provide a guarantee of success. Participants felt that their physical tear would not resolve with physiotherapy. As a meniscal tear had a clear damage to a structure, participants believed that surgery was the only option to resolve the physical damage. They could not see how physiotherapy could improve the symptoms or repair the joint. *‘The inside of your knee is, is very badly worn. And, however amount of physio you do, you’re not gonna sort of replace, you’re not gonna repair a damaged joint.’* (Participant 6)

## Discussion

This study describes the experiences and expectations of treatment pathways for meniscal tears in patients aged 18–55 years. Several themes were identified which focused on symptoms, the initial clinical consultation and the experiences of treatment. These themes were similar to the themes identified by authors of recent studies,^10 11^ which identified the importance of the MRI scan, the impact of meniscal tears of patient lives, the role of shared decision-making and also patient perceptions on the effectiveness of surgery and physiotherapy. This study strengthens existing themes and describes new themes which are described in more detail below.

The short-term impact of a meniscal tear has been previously described,^10 11^ however, this study described the medium-term and long-term effects. Participants described the impact the symptoms had on family life, occupation, emotional and financial well-being. Meniscal tears often affect individuals of working age with dependents, therefore, it is important for clinicians to understand if a patient is making a treatment decision based on factors such as impact on family life or finances.^16^ Studies in other musculoskeletal conditions have found that patients developed symptoms of a depressive disorder following an injury.^17^ Patients with a meniscal tear described the emotional impact their condition and treatment had on them, therefore, it is important that clinicians consider this and offer psychological help where required.

Participants strongly felt that the primary care doctor was the gateway into secondary care and the management of their condition would take place in secondary care by an orthopaedic surgeon. This could be due to the fact that all patients included were recruited from secondary care which is discussed further in the limitations. Nonetheless, this is contrary to the evidence which suggests that 47% of patients with a meniscal tear attend an outpatient and 22% undergo surgery.^18^ This suggests that the majority of patients with a meniscal tear can be managed by a primary care doctor.^18^ This qualitative study in combination with previous research highlights that clinicians should work with patients as comanagers of their health in order to change these preconceived ideas on treatment.

Participants believed that surgery provided definitive treatment and physiotherapy was likely not to be as successful. However, there is no existing evidence that surgery is more effective than physiotherapy. Current treatment guidelines recommend urgent operative treatment for bucket handle tears, for the remainder of tears a period of non-operative management is recommended.^4 5 19^ It is important during the initial clinical consultation to inform patients of existing evidence and highlight the role of physiotherapy in reducing symptoms and functional limitations.^4 5 19 20^

Patient perceptions may also be informed by the experience of peers who may or may not have had surgery for their meniscal tear. This could explain why patients were strongly in favour of meniscal repair compared with debridement. Meniscal repairs are typically for those with acute traumatic tears and have a failure rate close to 20%.^21^ Meniscal repair is typically reserved for patients who are younger with traumatic tears.^5^ It is important to state during the initial consultation that a meniscal tear is an umbrella term encompassing a wide variety of tear patterns and symptoms. This leads to tears requiring different management and not all tears being treated the same way. In particular, not all tears require repair and are repairable. This should in turn prevent patient expectations of specific treatments based on the experiences of their peers.

This study included patients with all types of meniscal tears and injury mechanisms to obtain a broad understanding of patient expectations and views on the treatment pathway. As a result, patients with both traumatic and degenerative meniscal tears were included. Research evidence and guidelines describe different treatments required for these different tear types.^5 19^ Having patient with different tear types could lead to different treatment expectations. For example, younger patients with traumatic meniscal tears may want to return to sport or high-energy activities much earlier. It could be argued that this is a limitation of this study as all patients with a meniscal tear have been grouped together. Based on the purposive sampling, the mean age of our sample was 42 with 6 patients not recalling a specific injury. This is suggestive of potentially degenerative tears, which may have a different symptom profile and require different treatment. Further studies may be required in exclusively younger patients with a history of trauma.

This study did not include data on the physical activity of different participants including their sporting background. Those who take part in physical activity may be more likely to push for operative intervention with the aim of returning to sport. Subgroup analysis on traumatic and degenerative tears or based on physical activity was not performed and there is scope for further work in this area. The purpose of this work was to add to the existing literature which contains few studies. This study has produced new and additional themes to the existing literature and increases the knowledge base on patient experiences of a meniscal tear.

A limitation of this study’s methodology was the lack of member checking and participant validation of the themes generated. As mentioned above, this is one of the few studies on patient experiences of meniscal tears and will contribute new themes to the literature. Further studies with member checking will allow consolidation and addition to these themes. There was also little data supporting non-operative treatment which is an effective and established treatment for these patients. This could be because the interview schedule was focused towards surgery, however, the researchers were aware of this potential bias before starting and the interview schedule was reviewed by a qualitative researcher. Second, the themes generated were similar to those identified in the two previous qualitative studies on patients with a meniscal tear.^10 11^ Another reason for this limitation could be because participants were recruited from secondary care, all of the patients had been through primary care and were being reviewed by an orthopaedic surgeon in a secondary care setting. This could influence the themes generated as these patients all likely have been through a primary care and some form physiotherapy setting. The results may not be fully generalisable to patients who are managed solely in primary care successfully with non-operative interventions. Future research focusing on meniscal tear management in primary care is required.

The strengths of this study include the use of purposive sampling, which ensured there were data from individuals of different age, gender and treatment groups. By taking a sample of participants from the METRO cohort study, it ensured participants at different points of the treatment pathway were included, this increased the breadth of the experiences described in this study. In addition, peer coding was performed for four of the interviews, this further increased the breadth of codes and themes generated from the data.

## Conclusion

There is a disconnect between patient experience and expectations and the existing scientific evidence. Up-to-date information, individualised for the patient is required for patients to ensure they are informed in their decision-making. Clinicians should be aware of patient experience, as this study demonstrates that patient perceptions may differ from accepted wisdom from other aspects of the literature, and both are needed to effectively guide patients in a shared decision-making process.

## supplementary material

10.1136/bmjopen-2024-088656 online supplemental file 1

## Data Availability

Data are available on reasonable request.
