# Supplementary material for: ‘Obviously, because it’s a tear it won’t necessarily mend itself’: a qualitative study of patient experiences and expectations of treatment for a meniscal tear
Source: BMJ Open. 2025 Jan 14;15(1):e088656. doi: 10.1136/bmjopen-2024-088656 (PMC11752057; doi:10.1136/bmjopen-2024-088656)
Supplement: online supplemental file 1 [file bmjopen-15-1-s001.pdf]

Appendix 1:

A copy of the interview schedule used as the guide to perform the semi-structured interviews.

Interview Schedule: METRO interview study

Introduction

Hello my name is Imran, a Doctor and researcher from Warwick University.
As you know, we are conducting a research study linked to the METRO study which you are already participating in.
You have been given an information sheet, the opportunity to ask questions and sign a consent form specific to this study – The METRO interview study. I would also like to remind you that you can withdraw from this study at any time during the interview and up to 2 weeks after the completion of this interview.
During this interview I would like to ask you about what its like to live with a meniscal tear and your experience of this injury. I would also like to ask about one of the questionnaires commonly used in research studies and your views on future research studies on meniscal tears. It is important to note that there is no right or wrong answers here, we are just exploring your experiences of your meniscal tear.
Can you please confirm for your recording that you understand this and are willing to continue?

Part 1: Experiences of meniscal tear

- Could you start by telling me about your meniscal tear?
- Further probes:
    - Was there a specific injury that brought it on?
- How did the symptoms affect your daily life?
    - How did it affect your ability to get around?
    - How did it affect your ability to perform routine daily tasks?
    - How did it affect your ability to work?
    - Did it affect your ability to play sport?

For each question above follow up with probing question of 'how did it make you feel'

- How did you go about dealing with your injury?

    - Did you have any idea what had happened?
    - Did you do some research into treatments?

Part 2: Seeking clinical help

- Can you tell me why you sought medical help and what that was like for you?
    - Was there anything that specifically led to that decision?
- What were your views on the initial consultation?
    - Did it match your expectations? What did you expect from the initial meeting?
- Were there any tests you expected?
    - What was your experience on the MRI results being shared with you? Once you heard you had a meniscal tear what went through your mind?
- Did you see an orthopaedic specialist? If so what was your experience of this?
- What were your thoughts on the treatment options offered?
    - Did you have a preference?
    - Could you tell me a bit more about your experiences of the treatment for your meniscal tear?
    - Did anything influence your choice of treatment? What were the important factors in your treatment choice?
    - Did you feel able to switch between one treatment choice and another?
- Tell me about your experiences following treatment?
    - How did it affect your life?
    - Were you happy with your treatment choice?

Part 3: Current outcome scores

You have completed some of the questionnaires for the METRO studies. These include questionnaires that are widely used in meniscal tear and knee research. I would like to focus on the WOMET questionnaire.
(provide patient with a copy of the questionnaire)

- What was your overall views this questionnaire?
- Do you feel it asked important questions?

Physical symptoms

Giving way or insecurity

- Was this something that bothered you?
- How important was this in your meniscal tear recovery?

Pain after activity

- Was this something that bothered you?
- How important was this in your meniscal tear recovery?

Range of motion

- Was this something that bothered you?
- How important was this in your meniscal tear recovery?

Numbness in and around your knee

- Was this something that bothered you?
- How important was this in your meniscal tear recovery?

Stiffness in your knee

- Was this something that bothered you?
- How important was this in your meniscal tear recovery?

Weakness in your knee

- Was this something that bothered you?
- How important was this in your meniscal tear recovery?

Swelling in your knee

- Was this something that bothered you?
- How important was this in your meniscal tear recovery?

Sharp pain after full weight bearing

- Was this something that bothered you?
- How important was this in your meniscal tear recovery?

Cracking grinding or popping in your knee

- Was this something that bothered you?
- How important was this in your meniscal tear recovery?

<u>Sport/ recreation / work / lifestyle</u>

- How relevant were these questions to your injury and treatment?

Fear reinjuring your knee

- Was this something that bothered you?

- How important was this in your meniscal tear recovery?

Participation in pre injury activities

- Was this something that bothered you?
- How important was this in your meniscal tear recovery?

Squatting

- Was this something that bothered you?
- How important was this in your meniscal tear recovery?

<u>Emotions</u>

- How relevant were these questions to your injury and treatment?

Conscious of your knee

- Was this something that bothered you?
- How important was this in your meniscal tear recovery?

Worry about the future

- Was this something that bothered you?
- How important was this in your meniscal tear recovery?

Frustration due to knee

- Was this something that bothered you?
- How important was this in your meniscal tear recovery?

<u>Overall</u>

- How did you feel when completing this questionnaire?
- Do you feel that all of the included items are important to you and your recovery?
- Are there any questions which you don’t feel apply to you?
- Are there any questions which you didn’t want to answer?
- Are there any questions which you didn’t feel were relevant?
- Are there any questions which you believe were not covered in this questionnaire which would be important to explore?

<u>Part 4: Future research ideas</u>

Previous research has explored the success of different treatments and compared different treatments against each other. They have identified success either through questionnaires or imaging findings.

- Is there anything you feel would be important for us to research?
- Is there anything you would have changed about your experience in the METRO study?

Part 5: Closing comments

- Is there anything else you would like to comment on regarding the things we have discussed?

Many thanks for taking the time to share your views with me, we really appreciate it. Please do contact us if you have any questions regarding the study. My contact details are found on the patient information sheet.

Once again thank you for your help.
