# Supplementary material for: ‘Obviously, because it’s a tear it won’t necessarily mend itself’: a qualitative study of patient experiences and expectations of treatment for a meniscal tear
Source: BMJ Open. 2025 Jan 14;15(1):e088656. doi: 10.1136/bmjopen-2024-088656 (PMC11752057; doi:10.1136/bmjopen-2024-088656)
Supplement: Supplementary file 2 [file bmjopen-2024-088656.reviewer_comments.pdf]

PEER REVIEW HISTORY

BMJ Open publishes all reviews undertaken for accepted manuscripts. Reviewers are asked to complete a checklist review form and are provided with free text boxes to elaborate on their assessment. These free text comments are reproduced below.

ARTICLE DETAILS

Title (Provisional)

'Obviously because it's a tear it won't necessarily mend itself.' A qualitative study of patient experiences and expectations of treatment for a meniscal tear.

Authors

Ahmed, Imran; Khatri, Chetan; Dhaif, Fatema G; Hutchinson, Charles E; Parsons, Nicholas; Price, Andrew James; Staniszewska, Sophie; Metcalfe, Andrew

---

VERSION 1 - REVIEW

**Reviewer**	**1**
**Name**	**Holm, Pætur**
**Affiliation**	**University of Southern Denmark**
**Date**	**19-Mar-2024**
**COI**	**NONE**

Overal comments:

Thank you for providing me with the opportunity to read and review this work.

Generally, I applaud studies of this type, investigating care experiences in order to better understand clinical practice patterns and improve care pathways.

Although, the research question has clinical value, my main worry is that interview findings are not discussed appropriately regarding context and sample of interviewees. As I mention several times below, you have interviewed a distinct group of people with meniscal tears, that primary would be classified as degenerative meniscal tears. This notion is somewhat confirmed by 6/10 not being able to recall the injury event, while 2 others (fall during walk, foot caught in pothole) also are more akin to degenerative meniscal tears rather than forceful trauma on otherwise healthy meniscii. You end up including a very wide age-range of informants (21 to 54 years; median age 45 years). This could work, if you purposefully selected informants at different age-intervals (ranging from younger to older) and also discussed interview findings in the context of these different age-intervals. As you can see below, I believe that this is critical to the interpretation of findings. However, there is no arguments for the included age-range, nor is there any discussion of how age may interact

with injury and care experience and how findings should be interpreted in this light. With this, lumping care experiences of a 21-year old together with care experiences of a 54-year old without discussing the age-factor seems odd and does disservice to the data.

Background

The background does not readily set the scene for exploring care experiences for the specific target population that was included. Some landmark randomized controlled trials regarding effects of initial surgical vs. non-surgical care for meniscal tears in both younger (DOI: 10.1056/EVIDoa2100038, DOI: 10.1136/bjsports-2021-105059) and older (DOI:10.1001/jama.2018.13308, DOI: 10.1097/PHM.0000000000000209, DOI: 10.1056/NEJMoa1301408) populations are not cited.

Aims and hypothesis

I dont believe that a pre-specified hypothesis should be included in this qualitative investigation.

Methods

Participant selection

People at different ends of the age-interval of 18-55 might have very different experiences living with a meniscal tear. Whereas the injury ethiology of younger individuals typically entails a forceful knee trauma resulting in a meniscal tear, injury ethiology for older individuals (35-40+ years) might be vastly different. Here, the underlying cause of injury is often age-related degenerative changes to the meniscii that predisposes to meniscal tears through injury mechanisms that do not neccessarily stem from forceful trauma, but rather daily-life activities. With degenerative meniscal tears, the knee-injured individual is often not able to precisely recall the injury event.

Also, the perspectives of living with a meniscal tear and care experiences may be vastly different between younger and older individuals. Whereas younger individuals may perceive the tear as a bigger trauma to their lives, due to the absence from sport and worries about knee function, older individuals may not have the same aspirations with sport and may be more content with having a knee that is not fully functioning (this is often perceived as a natural part of aging). This may have big implications for care experiences and expectations.

Looking at table 1, 7 oof 10 informants are 40+ years and 6 of 10 do not recall a specific injury event, which hints at degenerative meniscal tears as primary injury mechanism. The injury mechanism underlysing “fall when walking” and “foot caught in pothole” also hints at degenerative mensicus related injury. The fact that 6 of 7 informants that are 40+ years were treated non-surgically is in adcordance with evidence and clinical practice patterns, where clinicians are more inclined to offer non-surgical management of meniscal tears to this age-group due to both evidence of equality of treatment effects (compared to surgery) but also due to patient preferences, expectations and the type of physical activity/sport they are aiming to return to. The care pathway for younger individuals with traumatic meniscal tears

(e.g., 15-25 years) has more emphasis on getting people back to pre-injury sport/physical activity levels and clinicians are more inclined to offer surgery as the most meaningful treatment option, despite surgery not clearly demonstrating superiority of effect over initial non-surgical treatment and optional later surgery.

Data analysis

The choice of including 10 informants and not more seems more pragmatic and pre-specified and not driven by data saturation aspects. Please clarify for more transparancy.

Rigor

There is no presentation of the research team and their backgrounds, including potential pre-conceived notions about the subject field and how assumptions based on background and clinical care experiences were mitigated in the coding and interpretation of interviews. Please provide this for more clarity.

Context

The context of this interview study is essential for interpretation of these findings. This includes (but is not limited to) the health care setting and the specific type of informants.

Results

Under theme 1 (#2: inability to perform previous activities), for some reason you state that the informats comprise a relatively young population. In the context of knee injuries, with a median age of 45 years, I would hardly say that this is a relatively young population of informants with meniscal tears. As argued previously, these informants likely consist of primarily people with degenerative meniscal tears, where the injury mechanim and impact on daily life is vastly different from a young person, e.g., between 15 and 25 sustaining a sports-related traumatic meniscal tear. There is no distinction between the findings from the interview with they youngest and oldest subset of the interview samle.

Discussion

Overall, I miss a discussion of the sample of individuals that were included in this qualitative study. This is a specific sample of mostly middle-aged (median age 45 years) individuals with presumably degenerative meniscal tears. This has major implications for the injury experience and perceptions and expecations of treatment. Omitting this important factor in the discussion is a significant drawback, severely affecting the clinical interpretation of the findings. A discussion of this could be included in a discussion section related to context. The current brief mentioning of context (secondary care) in the limitations section is not sufficient.

As such, the authors miss the chance to discuss how injury and care experiences may differ between different age-groups and what implications this has for care needs.

VERSION 1 - REVIEW

**Reviewer**	**2**
**Name**	**O’Leary, Helen**
**Affiliation**	**Health Service Exceutive, Physiotherapy**
**Date**	**16-Dec-2023**
**COI**	**NONE**

Interesting study in a research area where there is a lack of qualitative investigation. Results indicate much more work is needed here to implement current evidence into practice.

Background
It would help for clarity to make the distinction between traumatic and degenerative meniscal tears in the first paragraph, as clinically there are different entities and separate bodies of evidence for each. Linked to this (Line 7/8) I don't think there was any uncertainty that conservative or non-surgical management is first line treatment for degenerative meniscal tears (based on lack of superior outcomes from surgery in the medium and long-term), the evidence about acute meniscal tears in young adults is uncertain-(Skou et al, 2022)

BJSM have also produced clinical practice recommendations, patients were included in this panel. Importance to refer to these seeing as they were not produced by an orthopaedic society (Siemieniuk et al 2017)

Line 24: few qualitative studies for knee pathologies? A large number of knee OA qualitative studies. Consider rephrasing "few qualitative studies for knee pathologies, such as, meniscal tears

Methods
Inclusion/exclusion criteria
Without the reader having to consult the METRO protocol, could the authors indicate if patients with co-existing knee osteoarthritis were included or excluded

Interview questions- Present a table with a description of questions in the schedule or a brief description of the question schedule in the Methods.

Data Analysis. Has there been input from a researcher with expertise in qualitative research? More information needed on how the researchers ensured rigor and trustworthiness in their approach to qualitative analysis.

For example: How many coders were involved? Was there peer coding? Was there participant validation or member checking carried out?
Reflexivity- Who was the researcher? What was their professional background? Were participants aware of this background? What were their own biases and preconceptions and how did that impact on their decision making etc?

Results
In thematic analysis a 'category' is a phrase used to describe a group of codes. Unsure of its use here to describe a group of themes.

Theme 1 - patient experience of symptoms is described in a lot of detail, could this section be shortened, to some extent this broader impact is common across all musculoskeletal conditions. Condensing this could allow scope for a more in-depth discussion

Providing more detail about where patients are on their 'treatment pathway' would help put the quotes and results in a clearer context. For example in sub-themes 10, 12, 13 patients place emphasis on the importance of meniscal repair to aid recovery. Has the patient consulted with an orthopaedic surgeon and physiotherapist at this point?

Would be good to see the presentation of more opposing or alternate views. For most sub-themes there is consensus

Scant description of the experience of physiotherapy or exercise therapy or any other reference to conservative management. Would have been good to learn more about patients' experiences of physiotherapy to balance the Results section on experience and expectation of treatment, which is dominated by surgery.

Discussion

Interesting evidence presented in the Results section on the strong beliefs around meniscal repair that persists among this secondary care cohort, (the majority of whom likely have degenerative meniscal tears, with the exception of participant 100 and 130). Evidence suggests that meniscal repair is unlikely in this middle-aged cohort, with predominantly pain of insidious onset, yet the focus of participants is on the possibility of repair, despite presumably having met several health professionals up to this point on their patient journey. This raises potential questions about how clinicians are educating and communicating with patients in the consultation. It would have been valuable to see this discussed (e.g evidence dissemination in primary care/changing clinician behaviours) and any resulting clinical and research implications.

“This suggests that over 50% of patients with a meniscal tear can be managed by a primary care doctor” This statement suggests 50% should be referred to secondary care. Surely if surgery is reserved mainly for acute locked knee/bucket handle tear, the vast majority of degenerative meniscal tears (which make up the majority) can be managed in primary care?

Page 17- Participants strongly felt that the primary care doctor was the gateway into secondary care – this is likely to be the case in a sample selected from secondary care?

Line 32. Should read “experience of peers” not “in peers”

Page 17. Would have been good to see more acknowledgement that this sample contains two clinical entities (degenerative meniscal tears and acute meniscal tears). The authors do acknowledge that meniscal tear is an “umbrella term” but within the manuscript there is little distinction made that they merit different approaches , and that new evidence/RCTs are also emerging suggesting non-surgical management could also be first line for acute meniscal tears. For readers not overly familiar with this clinical area this would be helpful.

Strengths/Limitations section
What is the meaning of "Independent" data analysis and how does this ensure "appropriate" generation of themes?

VERSION 1 - AUTHOR RESPONSE

Reviewer: 1

Reviewer: 1

Dr. Pætur Holm, University of Southern Denmark

Comments to the Author:

Overal comments:

Thank you for providing me with the opportunity to read and review this work.

Generally, I applaud studies of this type, investigating care experiences in order to better understand clinical practice patterns and improve care pathways.

Although, the research question has clinical value, my main worry is that interview findings are not discussed appropriately regarding context and sample of interviewees. As I mention several times below, you have interviewed a distinct group of people with meniscal tears, that primary would be classified as degenerative meniscal tears. This notion is somewhat

confirmed by 6/10 not being able to recall the injury event, while 2 others (fall during walk, foot caught in pothole) also are more akin to degenerative meniscal tears rather than forceful trauma on otherwise healthy meniscii. You end up including a very wide age-range of informants (21 to 54 years; median age 45 years). This could work, if you purposefully selected informants at different age-intervals (ranging from younger to older) and also discussed interview findings in the context of these different age-intervals. As you can see below, I believe that this is critical to the interpretation of findings. However, there is no arguments for the included age-range, nor is there any discussion of how age may interact with injury and care experience and how findings should be interpreted in this light. With this, lumping care experiences of a 21-year old together with care experiences of a 54-year old without discussing the age-factor seems odd and does disservice to the data.

Thank you for reviewing this manuscript. We hope we have addressed each of your comments below. With regards to your comments above, the authors wanted to include the views of a wide range of patients presenting the secondary care with a meniscal tear of the knee. This would include both traumatic and degenerative tears. We used purposive sampling based on sex, age and type of treatment. This aim of this study was to explore patient views on treatment pathways and their expectations of treatment. The authors do not believe different tear types would influence this from the patient point of view.

Background

The background does not readily set the scene for exploring care experiences for the specific target population that was included. Some landmark randomized controlled trials regarding effects of initial surgical vs. non-surgical care for meniscal tears in both younger (DOI: 10.1056/EVIDoa2100038, DOI: 10.1136/bjsports-2021-105059) and older (DOI:10.1001/jama.2018.13308, DOI: 10.1097/PHM.0000000000000209, DOI: 10.1056/NEJMoa1301408) populations are not cited.

A paragraph has been added discussing previous research into clinical effectiveness and why patient experience data is important. Citations mentioned above have been included.

Aims and hypothesis

I dont believe that a pre-specified hypothesis should be included in this qualitative investigation.

The hypothesis has been removed.

Methods

Participant selection

People at different ends of the age-interval of 18-55 might have very different experiences living with a meniscal tear. Whereas the injury ethiology of younger individuals typically entails a forceful knee trauma resulting in a meniscal tear, injury ethiology for older individuals (35-40+ years) might be vastly different. Here, the underlying cause of injury is often age-related degenerative changes to the meniscii that predisposes to meniscal tears through injury mechanisms that do not neccessarily stem from forceful trauma, but rather daily-life activities. With degenerative meniscal tears, the knee-injured individual is often not able to precisely recall the injury event.

Also, the perspectives of living with a meniscal tear and care experiences may be vastly different between younger and older individuals. Whereas younger individuals may perceive the tear as a bigger trauma to their lives, due to the absence from sport and worries about knee function, older individuals may not have the same aspirations with sport and may be more content with having a knee that is not fully functioning (this is often perceived as a natural part of aging). This may have big implications for care experiences and expectations.

Looking at table 1, 7 oof 10 informants are 40+ years and 6 of 10 do not recall a specific injury event, which hints at degenerative meniscal tears as primary injury mechanism. The injury mechanism underlysing “fall when walking” and “foot caught in pothole” also hints at degenerative mensicus related injury. The fact that 6 of 7 informants that are 40+ years were treated non-surgically is in adcordance with evidence and clinical practice patterns, where clinicians are more inclined to offer non-surgical management of meniscal tears to this age-group due to both evidence of equality of treatment effects (compared to surgery) but also due to patient preferences, expectations and the type of physical activity/sport they are aiming to return to. The care pathway for younger individuals with traumatic meniscal tears (e.g., 15-25 years) has more emphasis on getting people back to pre-injury sport/physical activity levels and clinicians are more inclined to offer surgery as the most meaningful treatment option, despite surgery not clearly demonstrating superiority of effect over initial non-surgical treatment and optional later surgery.

Thank you for your comments. The purpose of this study was to explore patient experiences of treatment pathways and their expectations of treatment. We believe we have a range of patients ages, sex, mechanism of injury and treatment in order to address our aims. Whilst we agree some patients may have a degenerative meniscal tear in the absence of significant trauma their expectations of treatment and views on the existing treatment pathways still provide invaluable information. We are not trying to make inferences of one treatment being better than the other. As there is a lack of data in patients with a meniscal tear we believe a broad approach is needed in the first instance before further work can be carried out in a subset of patients with a meniscal tear.

Data analysis

The choice of including 10 informants and not more seems more pragmatic and pre-specified and not driven by data saturation aspects. Please clarify for more transparancy.

Further detail has been added.

After nine interviews saturation of data was reached with no new themes identified from the ninth and tenth interview. During the recruitment period, through purposive sampling 17 participants from the METRO cohort population of 200 were initially invited for interview. As ten participants agreed to be interviewed no further participants were invited for interview at that point. These 17 patients were selected based on sex, age, number of months since recruitment, duration of knee pain (months) and management of meniscal tear.

Rigor

There is no presentation of the research team and their backgrounds, including potential pre-conceived notions about the subject field and how assumptions based on background and clinical care experiences were mitigated in the coding and interpretation of interviews. Please provide this for more clarity.

A section on reflexivity has been added including the role of the lead interviewer and the impact this may have had on coding.

Context

The context of this interview study is essential for interpretation of these findings. This includes (but is not limited to) the health care setting and the specific type of informants.

A section on context has also been provided in the methods section.

Results

Under theme 1 (#2: inability to perform previous activities), for some reason you state that the informants comprise a relatively young population. In the context of knee injuries, with a median age of 45 years, I would hardly say that this is a relatively young population of informants with meniscal tears. As argued previously, these informants likely consist of primarily people with degenerative meniscal tears, where the injury mechanism and impact on daily life is vastly different from a young person, e.g., between 15 and 25 sustaining a sports-related traumatic meniscal tear. There is no distinction between the findings from the interview with they youngest and oldest subset of the interview sample.

We have removed the sentence suggesting it was a relatively young population. No subgroup analysis was performed based on different ages as the authors wanted to include a broad age range so that a depth of experiences could be identified.

Discussion

Overall, I miss a discussion of the sample of individuals that were included in this qualitative study. This is a specific sample of mostly middle-aged (median age 45 years) individuals with presumably degenerative meniscal tears. This has major implications for the injury experience and perceptions and expectations of treatment. Omitting this important factor in the discussion is a significant drawback, severely affecting the clinical interpretation of the findings. A discussion of this could be included in a discussion section related to context. The current brief mentioning of context (secondary care) in the limitations section is not sufficient.

As such, the authors miss the chance to discuss how injury and care experiences may differ between different age-groups and what implications this has for care needs.

Thank you this comment. We have added further information in the discussion highlighting this limitation. As mentioned above, rather than focus on degenerative vs traumatic meniscal tears we wanted to focus on the experiences of all patients with a meniscal tear to provide a broad description. We accept that with our purposive sampling the patients do have a mean age of 42 and 6/10 do not recall a specific injury suggesting they may potentially be degenerative tears. We have explained this in our discussion and limitations section.

Reviewer: 2

Dr. Helen O'Leary, Health Service Exceutive

Comments to the Author:

Interesting study in a research area where there is a lack of qualitative investigation. Results indicate much more work is needed here to implement current evidence into practice.

Thank you for your comments and feedback

Background

It would help for clarity to make the distinction between traumatic and degenerative meniscal tears in the first paragraph, as clinically there are different entities and separate bodies of evidence for each. Linked to this (Line 7/8) I don't think there was any uncertainty that conservative or non-surgical management is first line treatment for degenerative meniscal tears (based on lack of superior outcomes from surgery in the medium and long-term), the evidence about acute meniscal tears in young adults is uncertain-(Skou et al, 2022)

BJSM have also produced clinical practice recommendations, patients were included in this panel. Importance to refer to these seeing as they were not produced by an orthopaedic society (Siemieniuk et al 2017)

We have mentioned these guidelines in the introduction. These guidelines focus on degenerative knee conditions on the whole comparing arthroscopy (+/- meniscectomy) vs non-operative treatment. As these does not relate to the entire population of patients with meniscal tears. We did not discuss these guidelines in substantial detail.

Line 24: few qualitative studies for knee pathologies? A large number of knee OA qualitative studies. Consider rephrasing "few qualitative studies for knee pathologies, such as, meniscal tears

This has been rephrased.

Methods

Inclusion/exclusion criteria

Without the reader having to consult the METRO protocol, could the authors indicate if patients with co-existing knee osteoarthritis were included or excluded

Established radiographic OA was an exclusion criteria.

Interview questions- Present a table with a description of questions in the schedule or a brief description of the question schedule in the Methods.

We have provided a brief description in the methods and provided a full copy of the interview schedule in appendix 1.

Data Analysis. Has there been input from a researcher with expertise in qualitative research?

SS is a professor who specialises in qualitative research with an extensive background in patient experiences research. SS oversaw the entire study from conception through to production of this manuscript.

More information needed on how the researchers ensured rigor and trustworthiness in their approach to qualitative analysis.

Further detail has been added to the methods section in the reflexivity, context and analysis section.

For example: How many coders were involved? Was there peer coding? Was there participant validation or member checking carried out?

We have added detail on peer coding. Member checking or participant validation was not performed and we have added this our limitations. Thank you for this feedback.

Reflexivity- Who was the researcher? What was their professional background? Were participants aware of this background? What were their own biases and preconceptions and how did that impact on their decision making etc?

A reflexivity paragraph has been added in the methods section.

Results

In thematic analysis a ‘category’ is a phrase used to describe a group of codes. Unsure of its use here to describe a group of themes.

We have removed the term category and rephrased this sentence.

Theme 1 - patient experience of symptoms is described in a lot of detail, could this section be shortened, to some extent this broader impact is common across all musculoskeletal conditions. Condensing this could allow scope for a more in-depth discussion

Some of the themes in the patient experiences of a meniscal tear have been shortened to allow further detail to be added in the discussion.

Providing more detail about where patients are on their ‘treatment pathway’ would help put the quotes and results in a clearer context. For example in sub-themes 10, 12, 13 patients place emphasis on the importance of meniscal repair to aid recovery. Has the patient consulted with an orthopaedic surgeon and physiotherapist at this point?

All patients have been reviewed by an orthopaedic surgeon at this point. We have added detail in the methods section regarding where patients were in their treatment journey.

'Due to the cross-sectional nature of this interview study, some patients had been recruited from the very start of their treatment in secondary care whereas others may have completed their treatment journey.'

Would be good to see the presentation of more opposing or alternate views. For most sub-themes there is consensus.

We have presented some contrasting opinions in the arguments against surgery theme. However, based on the data in the interviews the majority of patients were strongly in favour of surgery as evidenced by the themes generated. It was difficult to present opposing views based on the data in the study.

Scant description of the experience of physiotherapy or exercise therapy or any other reference to conservative management. Would have been good to learn more about patients' experiences of physiotherapy to balance the Results section on experience and expectation of treatment, which is dominated by surgery.

As above this was largely the result of the data produced in the interviews. This could potentially be a weakness of the interview schedule, however, this was reviewed by a senior qualitative researcher and the themes were similar to previous research in this area. We have added this to our limitations in the discussion.

Discussion

Interesting evidence presented in the Results section on the strong beliefs around meniscal repair that persists among this secondary care cohort, (the majority of whom likely have degenerative meniscal tears, with the exception of participant 100 and 130). Evidence suggests that meniscal repair is unlikely in this middle-aged cohort, with predominantly pain of insidious onset, yet the focus of participants is on the possibility of repair, despite presumably having met several health professionals up to this point on their patient journey. This raises potential questions about how clinicians are educating and communicating with patients in the consultation. It would have been valuable to see this discussed (e.g evidence

dissemination in primary care/changing clinician behaviours) and any resulting clinical and research implications.

Further detail has been added to the discussion

Patient perceptions may also be informed by the experience of peers who may or may not have had surgery for their meniscal tear. This could explain why patients were strongly in favour of meniscal repair compared to debridement. Meniscal repairs are typically for those with acute traumatic tears and have a high failure rate in those with degenerative tears. It is important to state during the initial consultation that a meniscal tear is an umbrella term encompassing a wide variety of tear patterns and symptoms. This leads to tears requiring different management and not all tears being treated the same way. In particular, not all tears require repair and are repairable. This should in turn prevent patient expectations of specific treatments based on the experiences of their peers.

"This suggests that over 50% of patients with a meniscal tear can be managed by a primary care doctor" This statement suggests 50% should be referred to secondary care. Surely if surgery is reserved mainly for acute locked knee/bucket handle tear, the vast majority of degenerative meniscal tears (which make up the majority) can be managed in primary care?

This statement was based on the previous research mentioned in the discussion.

Patients believed that the primary care doctor was a gateway into secondary care, however, we know from previous research that patients can be managed successfully in secondary. Based on 1,700 patients with a meniscal tear we found that 50% present to secondary care and 22% undergo surgery. We have changed the statement to describe the previous research. We are not stating which patients should or shouldn't be referred to secondary care.

Page 17- Participants strongly felt that the primary care doctor was the gateway into secondary care – this is likely to be the case in a sample selected from secondary care?

This has been made clear in the discussions section.

Line 32. Should read “experience of peers” not “in peers”

This has been changed

Page 17. Would have been good to see more acknowledgement that this sample contains two clinical entities (degenerative meniscal tears and acute meniscal tears). The authors do acknowledge that meniscal tear is an “umbrella term” but within the manuscript there is little distinction made that they merit different approaches , and that new evidence/RCTs are also emerging suggesting non-surgical management could also be first line for acute meniscal tears. For readers not overly familiar with this clinical area this would be helpful.

Further detail has been added in the discussion section

Strengths/Limitations section

What is the meaning of "Independent" data analysis and how does this ensure "appropriate" generation of themes?

This has been clarified.

In addition, peer coding was performed for four of the interviews, this further increased the breadth of codes and themes generated from the data.
